# Pregnancy, Birthing, and Postpartum Experiences During COVID-19 in the United States

**DOI:** 10.3389/fsoc.2021.611212

**Published:** 2021-02-08

**Authors:** Sarah E. DeYoung, Michaela Mangum

**Affiliations:** ^1^ Disaster Research Center and Sociology and Criminal Justice, University of Delaware, Newark, DE, United States; ^2^ Disaster Research Center, Joseph R. Biden, Jr. School of Public Policy and Administration, University of Delaware, Newark, DE, United States

**Keywords:** pregnancy, birthing, infant feeding, COVID-19, postpartum, post traumatic

## Abstract

The research aims of this project were to understand the impact of the COVID-19 pandemic on pregnancy, birthing, and postpartum experiences in the United States. Our data include responses from 34 states within the US. Findings from our analyses indicate that higher perceived social support predicted higher scores of well-being, while higher scores of perceived loneliness predicted lower scores of well-being, and higher trauma predicted lower well-being measured as satisfaction with life. Qualitative data support these findings, as well as the finding that there were various sources of stress for respondents during pregnancy, birth, and the postpartum timeframe—particularly in terms of managing work/occupation obligations and childcare. Additionally, this research fills a gap in understanding infant feeding in emergencies. Respondents perceived that early release from the hospital reduced access to lactation support, and many respondents reported receiving free samples of breastmilk substitutes through a variety of sources.

## Introduction: Post-Disaster Traumas

In disasters and humanitarian emergencies, families are vulnerable to the physical and social impacts of the hazard. They experience challenges and stress associated with evacuation and relocation (Bland et al., 1997; Lange et al., 2004; Curtis et al., 2007) and long-term psychological trauma (Boscarino and Adams, 2008). Families with small children are particularly vulnerable in disasters because they are susceptible to injury, illness, and other risks during the hazard event (Baker and Cormier, 2014). Because of the nature of lockdowns and restrictions on social gatherings during the COVID-19 pandemic, we anticipated that newly postpartum mothers and caregivers would experience more challenges with mental health, particularly postpartum stress from the burden of managing a “work-life balance”. We include a brief literature review of the key issues related to birthing, pregnancy, infant feeding, and parenting in disasters and pandemics. We then present the data we collected and findings regarding decisions about infant feeding during the COVID-19 pandemic and ways in which loneliness, trauma, and social support are associated with ill- or well-being. Policy implications for this work include recommendations for improving support services for families with infants and young children during pandemics and emergencies.

Apart from the structural damage and physical harm caused by disasters and emergencies, crisis events also cause psychological and emotional distress due to economic loss, job loss, loss of family members or friends, displacement, and the overall disruption of normal routines and social networks (Osofsky and Osofsky, 2018; Dayal De Prewitt and Richards, 2019). Longer-term psychological impacts such as mental health issues and PTSD (post-traumatic stress disorder) may also impact individuals after a major disaster or emergency (Wickrama and Kaspar, 2007; Beaglehole et al., 2018). High levels of stress in families resulting from stressor events such as disasters can have significant impacts on family relations and functioning. For example, a large amount of the stress and trauma among children following a disaster can be attributed to the amount of parental stress children experience at home (Abramson et al., 2010; Pfefferbaum et al., 2016; Osofsky and Osofsky, 2018). The impact of parental stress on children can be especially important to consider when children have lost other social support systems such as schools and childcare programs (La Greca et al., 2010; Dayal De Prewitt and Richards, 2019). High family stress may also be a factor leading to other familial issues including increased family conflict and domestic violence (Wickrama and Kaspar, 2007; Pfefferbaum et al., 2016).

Post-disaster increases in partner violence and child abuse cases have been widely reported (Enarson, 1999; Weitzman and Behrman, 2016; Gearhart et al., 2018; Parkinson, 2019; Seddighi et al., 2019). Disasters can also result in increased family stress, along with other factors such as economic instability, substance abuse or a history of violence or exposure to violence that contribute to this rise in domestic violence (Goodman, 2016; Weitzman and Behrman, 2016; Seddighi et al., 2019). Sexual violence against women and girls is especially prevalent in post-disaster contexts (Weitzman and Behrman, 2016; Seddighi et al., 2019). Women and young girls may be more vulnerable post-disaster due to the loss of social support systems and protective services (Enarson, 1999; Enarson, 1999; Curtis, Miller, and Berry, 2000). Moreover, with this loss of social networks and access to protective services, many incidents of domestic violence and abuse against women and children go unreported and may be understudied (Curtis, Miller, and Berry, 2000; Parkinson, 2019; Seddighi et al., 2019).

In addition to the risks of violence and exploitation women may face after a disaster or emergency, women face many unique challenges to their mental and physical health. High levels of stress and symptoms of PTSD are likely to have a more severe impact on women, especially during pregnancy (Xiong et al., 2010; Sohrabizadeh and Khankeh, 2016). Stress can also create adverse outcomes for pregnancy and early child development. High exposure to a disaster followed by high levels of stress or PTSD while in the early stages of pregnancy could lead to higher rates of premature births and low birth weight babies (Eskenazi et al., 2007; Hamilton et al., 2009; Goodman, 2016). High levels of maternal stress experienced in the prenatal phase could also affect a child’s behavioral outcomes, such as causing higher levels of anxiety during early childhood (McLean et al., 2018; Zhang et al., 2018). Overall, women’s and pregnant people’s health and reproductive care are at a greater risk during disasters and emergencies due to the lack of access to proper services and facilities and increased attention towards the general welfare of those impacted by the disaster or emergency, thereby limiting the availability of resources and services specifically concerned with reproductive health and care (Nour, 2011; Goodman, 2016). Disaster and emergency scenarios can lead to more adverse complications at birth and higher risks of maternal and infant mortality (Akker et al., 2011; Nour, 2011; Goodman, 2016; Mallett and Etzel, 2018; Singh et al., 2018). Unsanitary conditions and environments could cause infections or disease transmission, putting mothers and infants at increased risk of health complications and illness (Akker et al., 2011; Nour, 2011; Goodman, 2016; Mallett and Etzel, 2018; Singh et al., 2018).

Post-disaster trauma and stress are associated with an increase in premature births and low birth weight babies (Weissman et al., 1989; Eskenazi et al., 2007; Hamilton et al., 2009; Antipova and Curtis, 2015). Disasters and emergencies may likewise cause significant problems for infant feeding and nutrition. For the first six months after giving birth, infants should be exclusively breastfed and should continue to be breastfed up until the age of one or beyond (Centers for Disease Control and Prevention, 2019). Breastfeeding decreases the risk of infants contracting infections by strengthening their immune systems (Gribble et al., 2011; Hipgrave et al., 2012; DeYoung et al., 2018a; Gribble, 2018). Formula and breast milk substitutes increase a child’s risk of illness and infections, especially in a disaster setting where clean water and supplies needed to sterilize bottles may not be easily accessible (Gribble et al., 2011; Hipgrave et al., 2012; DeYoung et al., 2018a; Gribble, 2018). Infant formula itself may contain bacteria or be expired (Barron and Forsythe, 2007; DeYoung et al., 2018a; Cho et al., 2019), which can lead to serious diarrheal illness that can result in an increase in infant mortality (Gribble et al., 2011; Gribble, 2018).

Despite the advantages of breastfeeding, some factors may inhibit women from breastfeeding during a disaster or emergency. These include loss of support systems, loss of lactation support services/counseling, stress from evacuation and displacement, lack of privacy, and the perception of decreased milk supply (Gribble et al., 2011; DeYoung et al., 2018a; DeYoung et al., 2018b). Another major factor that leads many mothers to resort to formula during a disaster is the excessive and imprudent distribution of infant formula by companies and humanitarian organizations as part of relief efforts (Gribble et al., 2011; Binns et al., 2012; DeYoung et al., 2018a).

In the US, the COVID-19 virus was first identified on January 20th in Seattle, Washington when a citizen returned from an international trip to Wuhan. Less than 2 weeks later, cases were reported in Illinois, California, Arizona, and Massachusetts (Jernigan, 2020). By March 1, cases had spread to Wisconsin, Oregon, Florida, Rhode Island, and New York (Lardieri, 2020). Throughout early March, cases quickly multiplied to states in the north- and south-east, eventually spreading throughout the mid- and south-west. By March 17th, every US state had confirmed cases of COVID-19, with West Virginia being the last state to confirm a case (Department of Defense, 2020). Starting in March, states began implementing stay-at-home orders and other social distancing requirements. Out of the 50 states, 42 implemented state-wide stay-at-home orders (Mervosh et al., 2020). During this time, public health facilities and hospitals began implementing strict health and safety policies to decrease risks of transmission. However, many of these policies and measures taken to reduce the spread of COVID-19 have caused additional challenges and concerns for many mothers and their infants.

Clinical research on transmission of COVID-19 in pregnant people and infants is evolving. Data from infants born in Wuhan, China indicated no evidence of vertical transmission from mother to neonate (Chen et al., 2020; Schwartz, 2020). Because of the new clinical information, healthcare systems and facilities had varied interpretations of risk for transmission to infants. After the pandemic began in the United States, guidelines from the CDC included strict recommendations about preventing infants from becoming infected (Centers for Disease Control and Prevention, 2020; Mintz, 2020). These guidelines included the separation of the mother from the infant, despite scarce evidence about transmission of COVID-19 from mother/parent to child, and scarcity of evidence that the risk of COVID-19 outweigh the benefits of breastfeeding (Tomori et al., 2020). Scholars in birthing and infant feeding research cautioned against making policy mistakes and decisions similar to those made in past outbreaks that led to adverse impacts for child and maternal health, drawing parallels between early medical guidance regarding breastfeeding, the HIV/AIDS epidemic, and COVID-19 (Gribble, et al., 2020). Another adverse impact of the pandemic on birthing families is the capacity for adequate medical care and support for birthing people and mothers due to hospitals becoming inundated with COVID-19 patients (Rocca-Ihenacho and Alonso, 2020). Another issue that emerged during the pandemic was the increase in aggressive marketing by infant formula industries (Cullinan, 2020). This is also common after disasters, yet there is less research on the social and behavioral aspects of disaster capitalism (see Klein, 2007)—specifically its impact on women, young children, and birthing and pregnant people.

## Research Questions

Given the findings that COVID-19 resulted in new hospital policies, uncertainty about risk, and new efforts by formula companies to engage in aggressive marketing, the research aims of this project were to understand the following in terms of maternal and postpartum experiences:

1. The relationships among social support, pandemic-related trauma and well-being during the pandemic.
2. How experiences of birthing, lactation, and infant feeding are impacted by the pandemic.
3. The impacts of managing family stress or work-life issues during the pandemic.

## Methodology

In July of 2020, we launched a web-based survey to gather primary data in the United States, using Qualtrics as our survey platform. For recruitment tracking, we created a systematic list of mothering and parenting pages on social media (Facebook) from across the country. Some respondents also shared the survey in their own email listserves. We used a spreadsheet to list the names of the groups, and 30 groups per region. Specifically, we created a recruitment list of 30 groups in each region: the Northeast, Southwest, Midwest, Southeast, Central. We also sought groups in Alaska, Hawaii, Puerto Rico, The US Virgin Islands, as well as national-level groups (not tied to geographic regions). We identified the groups by searching for “parenting,” “coronavirus concerns,” “moms,” “new moms,” and key words related to infant feeding such as “breastfeeding” and “breastfeeding resources”. We documented the size of each Facebook page (in followers) and whether the group was public or private. We also created a separate list of national level groups that focus on parenting, breastfeeding, birthing, and COVID-19 discussions. To respect group rules and privacy, we prioritized posting study recruitment to groups that were either listed as “public” or groups that one of us already belonged to because of her status as a parent. In our recruitment, we indicated that the study was focused on people who were pregnant or gave birth from the timeframe of Dec 2019 through the time of data collection (summer of 2020). Most of the respondents were in the postpartum timeframe when they took this survey, but some were still pregnant (see results section).

The Institutional Review Board at the University of Delaware approved this research protocol. Respondents received a $10 electronic gift card via email for their responses, and we did not match names or identifying information with quotes or responses. Our survey included 40 questions about pregnancy, birthing, infant feeding, disaster preparedness, perceived trauma, loneliness, social support, and other perceptions about experiencing the pandemic as a new parent. For pregnancy questions, we asked when they were due to give birth, where they gave birth (home, hospital, birthing center, or other), if respondents had evacuated for a hurricane or disaster in the last two years, and type of feeding for infants during the pandemic (breastmilk, breastmilk substitutes, pumped milk, solids, combination, or other). For the disaster preparedness item, we asked respondents “How prepared do you think your household is for a natural hazard event such as a hurricane, wildfire, earthquake, flood, tornado, or other event?” (Likert scale 1–5).

### Scales and Data Screening

To measure potential traumatic experiences in women, we used the abbreviated PCL (PTSD Checklist) Scale by Lang et al. (2012) and Price et al. (2016). For this study, the PCL items were designed to capture the potential impacts of birthing during a pandemic. There are four items in this scale that ask for frequency of avoidance of trauma reminders, being easily startled, experiencing negative thoughts and repeated unwanted memories (Likert scale 1–5, with 1 being “least frequent” and 5 being “extremely frequent”). To measure well-being, we used the 5-item Satisfaction with Life Scale (SWLS) by Diener et al. (1985); these items include questions about perceived life satisfaction. Scale reliability using chronbach’s alpha for the PCL in the current sample of respondents was α = 0.74 and α = 0.82 for the SWLS. The items to measure loneliness and social support were both on a 5 point Likert Scale and asked respectively: “Please rate how frequently you do/feel the following during the pandemic (from March until now) on a scale from ‘never’ to ‘always’: Feel lonely, or feel connected to a social support system.” All these scales were used in the linear regression, in which the predictive variables were loneliness (Likert scale as described above), social support (Likert scale as described above), and trauma (abbreviated PCL described above). The outcome/dependent variable was well-being (SWLS).

We also included two open-ended questions at the end of the survey asking respondents: “In one to two sentences, please describe what you have been doing for self-care or would like to be doing for self-care during the pandemic (can include but not limited to: tasks for mental health, physical health, social networks, routines, creative tasks or other activities)?” and “What else would you like to share with us about your experience during the COVID-19 pandemic?” For the two open-ended questions, we asked respondents the following: 1) “In one to two sentences, please describe what you have been doing for self-care or would like to be doing for self-care during the pandemic (can include but not limited to: tasks for mental health, physical health, social networks, routines, creative tasks or other activities)?” and 2) “What else would you like to share with us about your experience during the COVID-19 pandemic?” For the self-care codes, we identified the following eight themes: 1) Physical activities (includes exercise, hiking, yoga, sleep); 2) Social strategies for coping (contact with family, friends, or partner); 3) Creative activities (includes spiritual, entertainment, cooking); 4) COVID risks preventing self-care; 5) Not able to find time; 6) Medication (antidepressants or antianxiety) or therapy; 7) Taking a break from social media; and 8) Other (response does not fall under other categories). For the “What else” item, we identified 6 code themes: 1) Concerned about partner at delivery, medical checks, and hospital policy; 2) General isolation; 3) Stress caused by job and work-life issues; 4) Breastfeeding and infant feeding concerns; 5) Positive or gratitude perceptions; and 6) Other.

All statistical analyses were conducted using SPSS 26 (IBM, 2019). To check for valid responses, we filtered out responses with a response duration of less than 120 s for response time to the entire survey or incomplete responses (i.e. if they only responded to the first item of “agree to participate,” this respondent was filtered from analyses). For qualitative data, we read through the themes and agreed upon a codebook for them (Appendix A). We then independently coded one round and adapted the codes after a second round of consolidating codes and identifying additional themes (Saldaña, 2014). To check for inter-rater reliability for items described in Qualitative Results Section, we calculated the score agreements using the Kappa statistics in SPSS. The agreement for the coding of the question about self-care was Kappa = 0.838 and Kappa = 0.825 for the “What else would you like to share?” question. There were 210 initial responses. After filtering for complete and valid responses, we had 192 responses (caregivers and parents) available for analysis.

## Results

### Demographics

Respondents lived in 34 different states, with the majority coming from the six following states: Delaware (51), North Carolina (26), Alabama (13), California (12), Texas (8), and Maryland (6). The majority of respondents indicated to be between the age of 26–36 (76.32%, or 145), while 7.3% (14) were age 18–25, 15.79% (30) were age 37–47, and one respondent indicated to be age 59 or older. The income of respondents varied (Table 1). The race of respondents skewed heavily white (88%, or 178), with 5% (10) indicating Hispanic/Latinx, 2.48% (5) Asian or Pacific Islander, one Black respondent, and 2% (4) checked “prefer not to indicate” ethnicity. For education, the sample indicated a higher education level than the general population (United States Census Bureau, 2020) (Table 2).

**TABLE 1:** Respondents’ level of reported income.

Level of income	Frequency	Percentage
10,000 or less per year	1	0.53
11,000–20,000	5	2.63
21,000–40,000	13	6.84
41,000–50,000	7	3.68
51,000–80,000	39	20.53
81,000–100,000	37	19.47
101,000–120,000	24	12.63
121,000–140,000	17	8.95
141,000–160,000	15	7.89
161,000 or higher	32	16.84
Total	190	100

**TABLE 2:** Respondents’ level of education.

Level of education	Frequency	Percentage
Less than high school	2	1.04
High school graduate	6	3.13
Some college	18	9.38
2 years degree	6	3.13
4 years degree	56	29.17
Master’s/Professional degree	58	30.21
Doctorate	46	23.96
Total	192	100

One Hundred and one respondents gave birth during the pandemic, while 15 gave birth before the pandemic began or were pregnant at the time of taking the survey. Of the respondents (who had given birth), 132 reported birthing at a hospital, and 5 at a birthing center, while 3 indicated a home birth. Notably, 37.59% (53) respondents indicated complications with their pregnancy or birth. These complications ranged from gestational diabetes, HELLP syndrome (hemolysis, elevated liver enzymes, low platelet count), intrauterine growth restriction, high blood pressure, polyhydramnios, and hemorrhaging. One respondent indicated that the fetus was nonreactive after a positive maternal COVID test, resulting in an emergency cesarean. The age of infants ranged from newborn to 12 months and older (Table 3). Thirty eight respondents indicated receiving a variety of support services (19.79%), including the Women Infants and Children (WIC) Supplemental Nutrition Assistance Program, unemployment benefits, and pregnancy Medicaid.

**TABLE 3:** Age of infants.

Age range	Frequency	Percentage
One-two months old	72	40.22
Three-five months old	71	39.66
Six-eight months old	20	11.17
Nine-eleven months old	3	1.68
Twelve months old or older	13	7.26
Total	179	100

### Quantitative Results

For questions regarding lactation and feeding, we asked respondents if they received infant formula during the pandemic in the form of free samples (they could indicate more than one method of receiving the free sample). A total of 92 respondents (35.8%) indicated that they received a free sample of infant formula in the mail; forty-one got the free sample at the pediatrician’s office (16%); and 36 (14%) received free formula at the hospital. Some respondents also indicated that they received formula from a friend or organization, or through an advertisement on social media. Sixty-five respondents (25.29%) reported “I did not receive a free sample.” In response to the question, “Were you always able to get the kinds of food you wanted to feed your infant/children throughout the pandemic?” many respondents indicated “Always” (117), Frequently (40), and Sometimes (23). Two respondents indicated that they were rarely able to get the kinds of food they wanted for their children. Respondents indicated that they received postpartum telehealth care (37), in-office visits (53), a mix of both for postpartum services (22), and some indicated that they did not request or need services (49). One respondent indicated that they were unable to afford postpartum care, and 5 indicated that they were unable to locate a provider of care for postpartum services. When asked if they received lactation support, 64.67% (97) indicated that the support they received was in the hospital or birthing center, and there were also respondents who needed lactation support but did not receive the needed services (Table 4).

**TABLE 4:** Lactation support after giving birth.

	Frequency	Percentage
Yes, in the hospital/birthing center	97	64.67
No, even though needed support	2	1.33
Yes, after leaving hospital	16	10.67
Did not want/need support	22	14.67
Other	13	8.67
Total	152	100

### Linear Regression

First, we conducted bivariate correlations on age, well-being, trauma, loneliness, and social support (Table 5). None of the variables were significantly correlated with age, which we excluded in the regression. To test for the impacts of pandemic-associated trauma, social support, and loneliness on maternal wellbeing, we conducted a linear regression analysis (see Table 6 for descriptive statistics). The overall model was significant (*p* < 0.005) and indicated that 25% (*R* ^2^ adj = 0.23) of the variance in well-being (*F* (3,172) = 19.29, *p* < 0.01) was predicted by perceived loneliness *b* = −0.172 (*p* = 0.27), *t* = −2.22 (negative direction); perceived social support *b* = 0.30 (*p* = 0.000), *t* = 4.10, and trauma *b* = −0.186, (*p* = 0.01), *t* = −2.60 (negative direction) (Table 7). In other words, in our model, higher social support predicted higher scores of well-being, while higher scores of perceived loneliness predicted lower scores of well-being, and higher trauma predicted lower well-being.

**TABLE 5:** Bivariate correlations.

	Q_age	Sum_trauma	Sum_well-being	Q_social support	Q__lonely
Q3_age					
r	1	−0.070	−0.009	0.072	−0.067
Sig. (2-tailed)		0.343	0.898	0.334	0.367
*n*	190	183	185	182	181
Sum_trauma					
r	−0.070	1	−0.314^a^	−0.244^a^	0.368^a^
Sig. (2-tailed)	0.343		0.000	0.001	0.000
*n*	183	183	183	179	178
Sum_wellbeing					
r	−0.009	−0.314^a^	1	0.415^a^	−0.371^a^
Sig. (2-tailed)	0.898	0.000		0.000	0.000
*n*	185	183	185	181	180
Q_socialsupport					
r	0.072	−0.244^a^	0.415^a^	1	−0.441^a^
Sig. (2-tailed)	0.334	0.001	0.000		0.000
*n*	182	179	181	182	179
Q__lonely					
r	−0.067	0.368^a^	−0.371^a^	−0.441^a^	1
Sig. (2-tailed)	0.367	0.000	0.000	0.000	
*n*	181	178	180	179	181

^a^ Correlation is significant at the 0.01 level (2-tailed).

**TABLE 6:** Means for regression, *N* = 176.

Variable	Mean	Standard Deviation
Well-being scale	19.0909	4.01038
Q loneliness	2.6648	0.97752
Q social support	3.0341	1.07383
Trauma scale	8.4489	3.38192

**TABLE 7:** Coefficients.

	Unstandardized coefficients		Standardized coefficients			95.0% confidence interval for B		Collinearity statistics	
Model	B	Std. Error	Beta	t	Sig.	Lower bound	Upper bound	Tolerance	VIF
(Constant)	19.417	1.502		12.931	0.000	16.453	22.381		
Q__lonely	−0.705	0.317	−0.172	−2.227	0.027	−1.330	−0.080	0.730	1.370
Q_social_support	1.127	0.275	0.302	4.104	0.000	0.585	1.670	0.804	1.244
Sum_trauma	−0.221	0.085	−0.186	−2.604	0.010	−0.389	−0.054	0.849	1.177

a. Dependent Variable: Sum_WB.

### Qualitative Results

Respondents indicated distress due to isolation, conflicting information about pregnancy and birthing and COVID-19, and stress on their families associated with the pandemic. Many respondents indicated several themes in one response. For example, if a respondent indicated that they were having concerns about breastfeeding and that they felt isolated after giving birth, we coded this as “General isolation” and “Infant feeding concerns.” Below we describe the themes that were most prevalent for respondents and provide example quotes from them. A major theme throughout these data were that respondents felt stressed about the isolation associated with the pandemic, despite their attempted coping mechanisms. To understand the ways in which respondents managed the isolation and stress, the self-care question and other open-ended questions provide a deeper insight to the findings from the regression analysis.

#### Self-Care

Respondents indicated that they engaged in self-care by doing physical activities such as walking or hiking. They also indicated keeping social connections with a small network of friends or family. Respondents also indicated that they use mental health or talk therapy, medication, and other means for managing their mental wellness. Many respondents indicated engaging in more than one form of self-care:

- *I practiced meditation and yoga throughout my pregnancy and I feel that that helped me cope well with going through the pandemic during my third trimester…/.. I go on socially distant walks with a few friends (who have also been socially distancing) and that has been very helpful.*
- *For self-care, my partner and I started therapy and I talk weekly to a friend and meditate.*
- *I started teletherapy and talk to my friends via text.*
- *I participate in a "Mommy Zoom" meeting every other week, chatting with my coworkers who are also on maternity leave.*
- *Going for walks, face-timing and talking to friends and family, doing puzzles, playing with my dog.../... Also, my husband has been going back to work more frequently now and I'm alone a lot. At first I enjoyed the alone time (it was self-care for me), but now I find it isolating.*

Some respondents also indicated that they do not have time for self-care or that they would like to do certain activities that they feel they cannot do because of the pandemic. For example:

- *I wish I could have more social interaction and my baby could be exposed to more close friends and family, however we are isolating.*
- *I have not been doing enough self care. Showers, podcasts while walking with the baby, and napping are probably my main forms of care right now.*
- *I would like to be able to take some time for myself and catch up on sleep.*
- *Would like to sleep and hike more and spend time in nature, sleep, sleep and have a clean house.*

These responses are related to some of the themes that we identified for the next portion of open-ended data. In these data, respondents indicated general stress, isolation, and frustration over their experiences with being pregnant and giving birth during the pandemic. We discuss these in order of themes and in order of the pregnancy, birthing, and postpartum timeframes.

#### General Stress

Pregnancy was a period of uncertainty for many respondents who felt frustrated by confusing or limited guidance from their healthcare providers. They indicated receiving conflicting or confusing information about hospital protocols and other health measures:

- *Pregnancy during a pandemic was stressful in a thousand big and small ways: the lack of data on outcomes for pregnant people and fetuses of a covid infection, going to prenatal appointments alone and masked, cancelled and telehealth appointments, cancelled prenatal classes, keeping up with changing labor and delivery policies.../...the collapse of in-person support networks, weighing the risks of going in for monitoring when something felt off or the baby wasn't moving much in-utero with the risk of covid exposure, and huge uncertainties...*
- *It’s been hard to go through 20* *weeks of pregnancy without really seeing family or friends, and we have a lot of questions about what our delivery in November is going to look like, and the support we will have with our newborn and toddler. It's been very frustrating for me and my OB, she feels like she doesn't have enough good information to give me guidance on staying safe during my pregnancy beyond the standard "social distance, mask, etc" recommendations.*

Additionally, the general stress the respondents described was not limited to one phase of pregnancy or birth—but rather throughout all phases of pregnancy, birthing, and caring for infants and toddlers. They also lamented missing out on rituals or celebrations associated with the arrival of the new infant (i.e. baby showers or other events).

#### Health Protocol Concerns

Many health protocol concerns centered around respondents’ hospital experiences and hospital policies related to COVID-19. In many cases, respondents had to choose between their planned support person such as a doula or their significant other or spouse. Some respondents indicated that they were worried about contracting COVID-19 before or during the timeframe of giving birth and that this might lead to separation from their newborn:

- *The hardest part of having my first baby in this was not being able to have my doula at the hospital then not being able to safely allow for some family members to visit following the birth.*
- *This is my third child. The birthing experience was different. It was hard to wear a mask during labor, I had been isolating so it felt terrifying that me or my baby could get COVID, the hospital shared that they would suggest separating Mom from baby if either tested positive (we didn't but this was also terrifying), and we were discharged exactly at the 24 h mark.*
- *The hospital birthing experience in NYC in March was scary with everything that was happening at that point. My husband and I weighed the options to deliver outside of the city or to be induced early. In the moment though, having a healthy birth and taking care of myself postpartum was most important there was little time or energy to worry about COVID in the hospital.*

Some respondents even indicated that they would not plan disclose to their healthcare providers if they experienced COVID-19 symptoms for fear of early labor inductions, separation from their infant, or some other forceful intervention. Again, some of these hesitations seemed to be related to the myriad of confusing information about birthing and hospital policies. This may also reflect a lack of trust in healthcare providers and the broader healthcare system.

#### Isolation

After they gave birth, many of the respondents described that their feelings of isolation made their postpartum experience more difficult. Many of their “usual” coping strategies were not possible because of the pandemic:

- *For me, having a newborn during the pandemic has been much harder than pregnancy was. We have taken social/physical distancing very seriously and have had no outside help or outsiders come (including family).../...It makes me sad that the only people who have ever held our baby or even touched her has been me and my husband and we don't know when we will feel comfortable having others interact with her safely.*
- *It’s extremely hard to deal with life with a newborn without the physical support of family and friends. I feel like I am on my phone more often just to feel some sort of connection and support.*
- *Family members have not yet met my daughter and she is almost 9* *weeks old. Two older relatives died of COVID-19. Two died of other causes, but we are unable to celebrate their lives with funeral services.*
- *The pandemic has caused tension and mistrust between me and family members. I feel I have to be extra cautious and question everything since I have a newborn baby.*

These quotes illustrate the combined impacts of isolation and the need to socially distance even from family members or friends that the respondents might normally see in person. Additionally, the feeling of the newborn as “fragile” seems to also be amplified because of the danger of COVID-19, which was reflected in the respondents’ comments about caution, risk, and decisions about social/physical distancing to keep the family safe.

#### Infant Feeding

Respondents indicated that the pandemic made their infant feeding choices and efforts more complex, especially because some were unable to receive in-person lactation support after they were discharged from the hospital:

- *Getting access to services postpartum has been difficult, especially with breastfeeding help. I have struggled mentally because I feel unsupported and isolated.*
- *I do think that the pandemic/lockdown contributed to my breastfeeding journey ending quicker than I wanted it to.*
- *Also because of the pandemic I was not able to receive lactation support after we left the hospital, which I believe contributed to my inability to breastfeed. Another factor that I believe contributed is that we were not able to have any family or friend support after the birth due to social distancing, and no childcare for our toddler, so we were stretched very thin and very stressed.*

In contrast, some respondents indicated that working from home facilitated their ease of maintaining their ability to continue to breastfeed or use a breast pump:

- *It has allowed for me to breastfeed (without pumping) and spend more time than expected with my infant which has been nice.*

Next, we discuss ways in which career and work obligations collided with taking care of the newborn, older children, and struggling to maintain well-being in daily life.

#### Work-Life Balance

Many concerns about work-life balance were related to lack of childcare for older children, to a sense of frustration in feeling unable to provide attention to all the children at once, and to stress about focusing on work-related tasks:

- *It has been extremely difficult to work from home while having a toddler in the house. Finding that balance has not gone well. I feel like I’m failing as an employee and as a mom.*
- *It is exponentially more stressful with an infant than it would have been if i didn't have a baby. Childcare was challenging to find when I had to return to work in the office. The unknowns about the virus are terrifying and the lack of responsibility and respect from the government towards families/parents with kids is horrible.*
- *It has been stressful going from a full time working mom to a mom staying at home with 2 children and trying to juggle ongoing work responsibilities, without the option to do what I would prefer to do when spending time with the kids: taking them out to the library, playground, zoo, pool, to visit friends, etc.*

The comments about balancing childcare suggest that the pandemic creates a unique situation in which new stress is generated by the pressure to continue performing and producing at work, or to appear as though “business is going on as usual.” Respondents also mentioned how normally they would include grandparents or extended family for assisting with childcare but that this was made more complex because of the pandemic.

#### Complex Feelings Mixed With Gratitude

Some respondents also indicated they had a lens of positivity, at least partially, for some of the new circumstances related to social distancing and being away from normal social interactions and routines. This theme does not minimize the severity of other adverse experiences, but rather reflects their attempts to cope during the pandemic:

- *I'm a pretty introverted person anyway so social distancing and quarantining isn't too hard for me to do. It helps that I love being home with my family as it is.*
- *It has been hard not being able to do things with my baby I would like to, especially holiday things. But I am also thankful for the extra time I have been able to be home (I am an elementary school teacher and never went back to work because of the pandemic, I taught online).*
- *There has been a lot of loss in this season, but we are full of gratitude for the privilege we have to be employed, supported (at a distance), and the ability to work from home full-time.*
- *It was sad not to have my family there with me to visit the baby, but there was also a strange peace about it only being my husband and I for two days we were in the hospital. I will note that the hospital staff were discharging mothers and babies as early as possible in efforts to keep people out of the environment due to the pandemic. We were out within 48 h.*

Although some respondents had a positive perspective on their experiences, many respondents indicated difficulties with coping, and this was reflected in much of the quantitative and qualitative data. Two respondents stated:

- *There is too much to include here. In short, the experience has been traumatizing.*
- *It has been incredibly frustrating to live in an area where lots of people are not taking covid19 seriously. My family has been chastised for not letting family members from outside the household visit while I was pregnant and especially now that we have a newborn at home.*

Together, these data and results suggest that most respondents experienced new challenges because of the pandemic. These challenges seemed to exacerbate the “usual” levels of stress, isolation, and other difficulties that new parents experience during the postpartum period. These findings were consistent across both the open-ended and the quantitative measures that we included for this study—particularly that social support mitigated the adverse impact of trauma.

## Discussion and Policy Implications

Pregnancy and birth during the pandemic were associated with anxiety and uncertainty for many of the respondents. Not being able to have their partner or support person at the hospital for birth—or having to choose between them—was a major point of concern. Respondents also indicated that release from the hospital was quick due to the new pandemic protocols. Additional research building upon these research findings should track the ways in which early release or shorter hospital stays impact outcomes for new parents and infants in a pandemic context or other major crisis event. One potential effect is that postpartum complications may go undetected—from difficulties with breastfeeding to life-threatening issues. There may also be other unanticipated mental health outcomes related to the shortened hospital stays. Some programs that carry out in-person home visits also had to adapt their protocols during the pandemic, which may have an impact on screening for postpartum depression, anxiety, and other postpartum mood disorders. Birthing in the pandemic may also increase other negative spill-over impacts related to postpartum care, because of reduced time in the hospital where many patients receive lactation and other support services.

For those respondents who felt disconnected from their social support system, the sense of isolation that many new mothers feel after the birth of their infants in normal times was exacerbated by the pandemic. Importantly, those who did feel connected to social support systems had higher levels of well-being. The postpartum period is a critical time for the new parent(s) to maintain mental health through social contact and social support systems (Tani and Castagna, 2017). However, because of the pandemic, many of the activities that the respondents wanted to do, especially attendance at social gatherings, meeting with other mothers and peers face-to-face, and other such were not possible for the parents who were self-isolating, quarantining, or adhering to guidelines put forth by health officials to mitigate the spread of COVID-19. Some respondents found creative ways to maintain social connections such as through taking outside walks or having outdoor visits with a friend or friends. However, it seems that these were not sufficient replacements for the kinds of social support and interaction that the respondents most desired.

One of our most alarming findings was that only approximately 25% of the respondents indicated that they did *not* receive a free sample of breastmilk substitutes (infant formula), while the remaining respondents did receive some form of complimentary or unsolicited infant formula sample. Often, respondents indicated that they received samples from more than one source. These sources included the mail, the hospital, the pediatrician’s office, or some other source, such as through social media or from a friend or peer. In regions where food scarcity and poverty were already prevalent before the pandemic, this aggressive marketing tactic has the dangerous potential to steer families away from breastfeeding (Rosenberg et al., 2008). Breastfeeding should most especially be supported during disasters and emergencies because it is a protective mechanism for infants and for the mother or lactating parent (see e.g. Gribble, 2018; Davis-Floyd et al., 2021). While it is not surprising that aggressive formula marketing is happening in the United States during the COVID-19 pandemic, additional research is required to understand to what extent the aggressive marketing “works” or becomes more effective at recruiting infant caregivers during times of crisis, disasters, and pandemics. Such additional research should include longitudinal studies that identify factors that bolster rates of exclusive and prolonged breastfeeding. Additionally, international research is needed in this area because policies and protections for breastfeeding in emergency scenarios do vary from country to country (Hoang et al., 2020).

The work-life balance concern for caregivers has received considerable attention in mainstream media, as well as in academic research. Unsurprisingly, in the United States in particular, women left the labor market during the summer and early autumn at significantly higher rates (Hsu, 2020). While may academic and research studies may center the changes and demands of working women, pregnant people, caregivers, and parents, it is important to understand how specific mechanisms associated with pregnancy, birthing, and postpartum care are associated with potentially severe outcomes such as postpartum psychosis, intersections of mental health with parenting and work, parenting and sense of community, and changes in social support systems because of other “spillover” effects of the pandemic such as family stress due to unemployment, COVID death/s in the family, and other complexities.

### Study Limitations

Our recruitment method was not a random sampling technique. However, it has been used to collect data rapidly after disasters (e.g. Mongold et al., 2020) to explore social and behavioral aspects of reactions in crisis scenarios. It is also difficult or impossible to conduct face to face interviews because of the contagious aspect of COVID-19. In future research, this systematic social media recruitment approach should be compared to other web-based recruitment techniques such as Lucid Theorem and Amazon Mechanical Turk. Partnering with a health organization or another nonprofit that has regular contact with pregnant patients and new parents would be a possibility for gathering future data, if patient/respondent privacy and autonomy is protected.

The fact that our survey sample was comprised of 88% white respondents is a serious limitation of our study. Women of color in the United States face greater health risks due to racism and discrimination in health care and lack of access to critical resources (Singh et al., 2017; Owens and Sharla, 2019). Hazards and disasters also disproportionately impact Black, Indigenous, and Hispanic populations (Davies et al., 2018). Given these aspects of vulnerability, the COVID-19 pandemic is likely to continue to adversely impact such families and result in furthering the gap between families who are thriving and families who experience barriers in accessing healthcare and support. Additional research should include measures for tracking these disparities over time and across race and ethnicity, as other scholars have already suggested (Lemke and Brown, 2020) and as other articles in this collection attempt to do.

Another limitation of this study is that the measure of the abbreviated PCL is arguably more appropriate for one event. The PCL for this study was designed to capture potential stress and trauma related to the pregnancy and birthing experience during the pandemic. The COVID-19 pandemic is a protracted crisis and not a single “point” in time. The complexity of the pandemic as an on-going event can make measurements more complicated compared to measuring stress associated with a rapid onset hazard event (such as a tsunami, earthquake, or other disaster).

## Conclusions: The Adverse Effects of Isolation and Stress and the Need for Their Mitigation

Our findings suggest that the isolation associated with the COVID-19 pandemic has adverse outcomes for maternal mental health, specifically psychological trauma during the postpartum time frame. This is not to say that social/physical distancing guidelines are not important, but rather that birthing and postpartum parents should be supported through social networks in new and creative ways. Many of the respondents reported that they found ways to continue socializing through virtual networks. These strategies for facilitating social interactions and social support networks should be considered by those working to provide care to families with infants and young children.

Additionally, stress associated with career and work-life balance should be mitigated through specific family-friendly policies at organizational and national levels. The United States still fails to provide adequate support for families because it does not have a national paid leave policy after birth (Nunez, 2020). The increased strain on families during the pandemic may also have adverse impacts on other indicators, such as abuse or neglect (Brown et al., 2020).

Similarly, the United States does not adhere to provisions set forth in the WHO Code, or the World Health Organization’s (1981) International Code of Marketing of Breastmilk Substitutes, making it easier for companies to encroach on healthcare facilities and other spaces that target new parents—as evidenced by the fact that many respondents received free infant formula samples during the pandemic. Organizations such as WIC, community-based organizations, and hospitals can and should generate more stringent internal policies that prevent aggressive formula marketing. Overall, the COVID-19 pandemic will likely have long-lasting adverse impacts on families, and these should be mitigated through evidence-based intervention programs.

## Data Availability

The datasets presented in this study can be found in online repositories. The names of the repository/repositories and accession number(s) can be found below: https://www.openicpsr.org/openicpsr/project/120802/version/V1/view.

## AppendixQualitative code manual

**Table:**

Self-care codes	
Physical activities (includes exercise, hiking, yoga, sleep)	1
Social (includes, family, friends, partner)	2
Creative, spiritual, entertainment, cooking	3
Not able to find time	4
COVID risks prevent self-care	5
Other	6
Medication or therapy	7
Alone time/breaks from media	8

Else Code	
Concerns about partner at delivery, med checks, and hospital policy	1
Isolation	2
Work balance and childcare	3
Lactation or feeding concerns	4
Positive or gratitude	5
Other	6
